# DiCo-EXT: Diversity and Consistency-Guided Framework for Extractive Summarization

**DOI:** 10.3390/e28010088

**Published:** 2026-01-12

**Authors:** Yiming Wang, Jindong Zhang

**Affiliations:** 1 College of Computer Science and Technology, Jilin University, Changchun 130012, China; ymw21@mails.jlu.edu.cn; 2 Key Laboratory of Symbolic Computation and Knowledge Engineering of Ministry of Education, Jilin University, Changchun 130012, China

**Keywords:** extractive summarization, redundancy reduction, semantic similarity consistency (SSC), diversity penalty loss, ROUGE evaluation limitations

## Abstract

ROUGE is a common objective for extractive summarization because n-gram overlap aligns with sentence-level selection. However, models that focus only on ROUGE often choose sentences with similar content, and the resulting summaries contain redundant information. We propose DiCo-EXT, a training framework that integrates two new loss terms into a standard extractive model: a semantic consistency term and a diversity penalty. The consistency module encourages selected sentences to stay close to document-level meaning, and the diversity penalty reduces semantic overlap within the summary. Both components are fully differentiable and can be optimized together with the base loss, without extra heuristics or multi-stage post-processing. Experiments on CNN/DailyMail, XSum, and WikiHow show lower redundancy and higher lexical diversity, while ROUGE remains comparable to a strong baseline. These results indicate that simple training objectives can balance coverage and redundancy without increasing model size or architectural complexity.

## 1. Introduction

Extractive text summarization builds a summary by directly selecting sentences from the source document, which naturally preserves factual content [1]. This property is important in domains such as healthcare, biomedicine, and law, where readers need statements that can be checked against the original text [2,3,4]. In multi-sentence news-style summaries such as those in the CNN/DailyMail corpus, redundancy between selected sentences is a recurring issue: models often pick several sentences that restate the same facts with slightly different wording. Most systems are trained and evaluated with ROUGE [5], which measures n-gram overlap with human-written references, so many recent models treat ROUGE as the main optimization target [6,7]. In practice, maximizing n-gram overlap can increase redundancy: the model tends to select sentences that repeat similar information, which raises within-summary similarity and wastes reading effort [8,9]. Standard sequence-labeling models [10] mainly score sentences in isolation and lack an explicit objective that shapes the selected set as a whole. Post hoc methods such as MMR [11] can filter redundancy but are decoupled from training, and graph-based approaches [12,13] introduce extra complexity and sensitivity to graph construction. For extractive summarization to be useful in real applications, the learning objective should therefore go beyond content recall and take both informativeness and diversity into account.

To address this gap, we propose a training-time framework, DiCo-EXT, that guides sentence selection through differentiable objectives rather than post hoc filtering. The framework introduces two complementary components: a Diversity Penalty loss $L_{div}$ and a Semantic Similarity Consistency (SSC) module. Together, they aim to balance informativeness and diversity while avoiding the limitations of heuristic or graph-based methods. The Diversity Penalty explicitly discourages high pairwise semantic similarity among candidate sentences, providing an end-to-end differentiable signal at the summary-set level. Unlike post hoc heuristics [11], it is directly aligned with the training objective, and unlike graph-based designs [12,13], it requires no auxiliary structures or additional hyperparameters.

Our ablation studies (Section 4) show that this formulation effectively balances the ROUGE–diversity trade-off. The Diversity Penalty substantially improves diversity metrics (e.g., lower Self-BLEU and higher Distinct-n) while causing only a minor decrease in ROUGE. These results suggest that content fidelity and diversity are inherently competing objectives, but can be jointly optimized to a balanced state that minimizes redundancy without losing key information. Moreover, they indicate that part of the gain in ROUGE observed under ROUGE-only training may stem from redundant content selection. We evaluate DiCo-EXT on three standard benchmarks—CNN/DailyMail (CNNDM), XSum, and WikiHow and find that it produces less redundant summaries with notably higher diversity, while maintaining competitive ROUGE performance. This demonstrates that DiCo-EXT provides a simple yet robust, fully trainable alternative to complex graph-based or reinforcement-learning approaches. Unlike prior systems that score sentences independently or apply redundancy control after selection, DiCo-EXT integrates both faithfulness and diversity within a unified differentiable framework. We evaluate DiCo-EXT on the CNN/DailyMail dataset and further test its generalization on XSum and WikiHow. CNN/DailyMail provides multi-sentence news summaries where redundancy between selected sentences is a central issue, while XSum and WikiHow represent different summary genres and compression levels. The main contributions of this work are:

- We propose DiCo-EXT, a unified training framework that jointly optimizes informativeness and diversity through differentiable objectives, addressing the redundancy issue inherent in ROUGE-based extractive summarization.
- We design an SSC module together with a Diversity Penalty to jointly preserve semantic faithfulness and reduce redundancy in extractive summarization.
- We validate DiCo-EXT on CNN/DailyMail, XSum, and WikiHow, showing improved diversity and reduced redundancy while maintaining competitive ROUGE scores.

## 2. Related Work

Extractive summarization has evolved through several methodological phases, progressing from surface-level heuristics to neural architectures. Early approaches exploited statistical and positional cues such as word frequency, sentence length, or location within a document [14,15,16]. Graph-based algorithms subsequently modeled sentence centrality using lexical or semantic similarity networks, providing a global perspective on document structure. While these models were computationally efficient, they primarily captured shallow lexical relations and struggled with deeper semantic representation. The emergence of neural architectures reframed extractive summarization as a sequence-labeling problem, where each sentence is assigned a binary salience label. Recurrent neural networks introduced the ability to model inter-sentence dependencies, and later, Transformer-based encoders offered superior long-range contextualization. The advent of pre-trained language models, notably BERT and its variants [17], further improved representation quality and quickly became dominant baselines. In addition, hybrid methods have explored integrating syntactic, discourse, or topic-level cues [3,12], while hierarchical encoders and attention mechanisms have enhanced document-level coherence. Despite these advances, most systems still rely on independent sentence scoring and lack an explicit objective that governs the collective diversity and faithfulness of the selected set.

The evaluation methodology has also played a central role in shaping research directions [18,19,20]. ROUGE [5] remains the de facto standard due to its reproducibility and interpretability, offering an efficient way to quantify overlap with human references. However, its reliance on n-gram matching has drawn criticism for rewarding lexical similarity rather than semantic completeness or non-redundancy. To compensate, redundancy-aware strategies such as Maximal Marginal Relevance (MMR) [21], graph-based extractive frameworks [12], and reinforcement learning approaches [22,23] have been proposed to encourage diversity during selection [24,25]. While effective to some extent, these models often require complex architecture design, sensitivity to hyperparameters, or post hoc filtering procedures, limiting their practicality. A growing body of work has since focused on semantic similarity modeling and diversity optimization as intrinsic training objectives. Embedding-based evaluation metrics [26] aim to better correlate with human judgments by assessing semantic coverage instead of raw token overlap. Parallel progress in contrastive learning [27,28] and representation regularization [29,30] has inspired methods that explicitly separate overlapping sentence embeddings and promote dispersion in semantic space. Other approaches introduce multi-task frameworks (e.g., joint keyword extraction or discourse prediction) and redundancy-regularized attention mechanisms to unify relevance and diversity within end-to-end training. More recently, LLM-based summarization systems (e.g., GPT-4- and Claude-style models) have also been explored for extractive selection or as components in hybrid pipelines. Compared with classical extractive models, LLMs often provide strong zero-shot capability and flexible instruction following, but they can be costly to run, harder to reproduce, and less transparent in terms of controllable redundancy behavior. In contrast, DiCo-EXT focuses on a lightweight and fully differentiable training objective that explicitly targets redundancy control while preserving semantic alignment, making it easier to train, deploy, and reproduce at scale. These directions are therefore complementary: DiCo-EXT can serve as a redundancy-aware extractor that supplies a compact, diverse evidence set for downstream LLM rewriting, while LLMs can provide pseudo-labels or preference signals to guide the training of efficient extractive selectors. We discuss these integration opportunities further in the Future Work section.

Overall, these research efforts converge toward the view that effective extractive summarization should jointly optimize informativeness, faithfulness, and diversity, rather than treating them as independent objectives. However, a lightweight and differentiable formulation that integrates semantic alignment with redundancy control remains underexplored—this gap motivates the DiCo-EXT framework proposed in this study.

## 3. Methodology

### 3.1. Overview

The architecture of the DiCo-EXT model is designed to directly address redundancy and semantic inconsistency in extractive summarization. Unlike multi-task learning frameworks that incorporate auxiliary objectives such as keyword extraction, DiCo-EXT adopts a streamlined single-task architecture and focuses on optimizing the collective properties of the selected sentence set. Specifically, it operationalizes the balance between informativeness and diversity through two differentiable loss terms, which shape the semantic space of sentence representations and encourage summaries that are both semantically coherent and non-redundant. However, despite progress in neural extractive summarization, most training objectives remain ROUGE-centric and sentence-wise, optimizing relevance to oracle labels without explicitly constraining the set-level behavior of the selected sentences.

As a result, models can achieve high ROUGE by selecting multiple semantically overlapping sentences, producing redundant summaries and creating a mismatch between the optimization target and the desired properties of an extractive summary. This reveals a precise research gap: a lightweight, fully differentiable objective that jointly enforces (i) semantic alignment for content coverage and faithfulness, and (ii) redundancy control within the selected set remains underexplored. To bridge this gap, DiCo-EXT introduces two complementary components: a Semantic Similarity Consistency (SSC) objective to preserve document–summary semantic alignment, and a Diversity Penalty to explicitly discourage similarity among selected sentences. Together, they enable end-to-end optimization of informativeness and diversity without post hoc heuristics.

**Figure 1:**
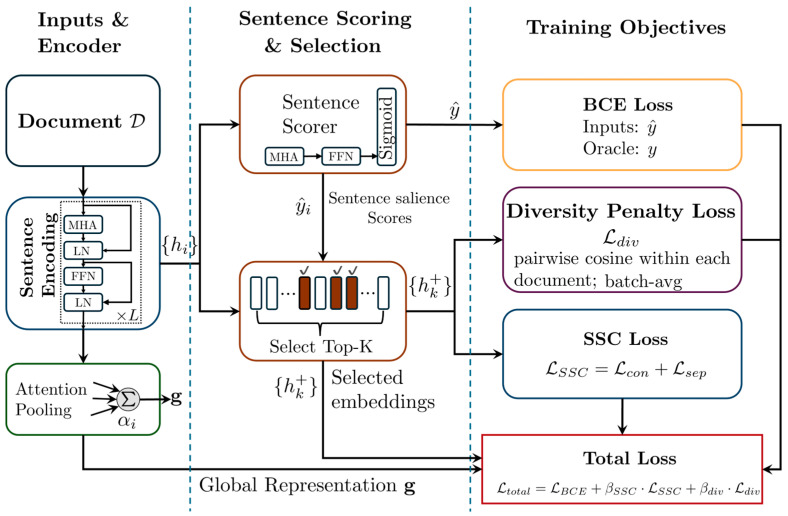
Overview of the DiCo-EXT framework.

As illustrated in Figure 1, the model processes a document through a shared encoder to obtain contextualized sentence representations. Figure 1 summarizes the overall training pipeline of DiCo-EXT. The document is encoded into contextualized sentence embeddings $h_{i}$, which are scored to select summary candidates. We further compute a document-level representation g via attention pooling. The model is optimized with three complementary objectives: $L_{BCE}$ for sentence selection, $L_{SSC}$ (including $L_{con}$ and $L_{sep}$) to align selected sentences with g while discouraging intra-summary collapse, and $L_{div}$ to penalize high cosine similarity among selected sentences and improve overall diversity. These representations are then scored by a classifier. The key improvement lies in the optimization process. In this step, the standard binary cross-entropy loss used for sentence selection is extended with two additional terms, the SSC loss and a diversity penalty. These components act directly on the semantic embeddings of the sentences selected during each training step.

### 3.2. Problem Formulation

Given an input document $D=s_{1},s_{2},\ldots ,s_{N_{d}}$ with $N_{d}$ sentences, extractive summarization selects a subset Y⊆D as the final summary. Most neural extractive models treat this task as sentence-level prediction and use ROUGE-based supervision during training. ROUGE is computed from n-gram overlap. As a result, it is not sufficiently sensitive to redundancy. Even if the model selects several semantically similar sentences, ROUGE can remain high, although little new information is added. This creates a gap between ROUGE-oriented optimization and the target summary properties, namely high coverage and low repetition. To reduce this gap, DiCo-EXT extends the standard objective with two differentiable embedding-level terms: $L_{\mathrm{SSC}}$ (Semantic Similarity Consistency) and $L_{\mathrm{div}}$ (Diversity Penalty). These terms promote semantic alignment while explicitly penalizing redundancy. Table 1 lists the principal symbols and their definitions used throughout Section 3.

### 3.3. Sentence Encoding

Let the input document be $D={{\{s}_{i}\}}_{i=1}^{N_{d}}$, which contains $N_{d}$ sentences. For each sentence $s_{i}$, we first tokenize the text and prepend the special token $\left[\mathrm{CLS}\right]$. We then feed the resulting token sequence into a pre-trained Transformer encoder (e.g., BERT) to obtain contextualized token embeddings. The sentence representation of $s_{i}$ is taken from the encoder output at $\left[\mathrm{CLS}\right]$, and is further transformed by a non-linear projection:

(1) $$h_{i}=\sigma \left(W_{p}\cdot \mathrm{Encoder}{\left(s_{i}\right)}_{\left[\mathrm{CLS}\right]}+b_{p}\right)$$

Here, $\sigma \left(\cdot \right)$ denotes a non-linear activation that increases the expressive capacity of the projection. The parameters $W_{p}\in R^{d_{h}\times d_{e}}$ and $b_{p}\in R^{d_{h}}$ are learnable. The value $d_{e}$ is the encoder output dimension, and $d_{h}$ is the target hidden dimension. The resulting vector $h_{i}\in R^{d_{h}}$ is used as the semantic representation of the sentence $s_{i}$.

### 3.4. Semantic Similarity Consistency (SSC) Module

This module is responsible for calculating the SSC loss, which enforces two critical properties in the selected summary: intra-summary consistency with the document’s main themes and inter-sentence distinction to avoid redundancy.

#### 3.4.1. Document Representation

To compute a global document representation g, we use a weighted average of all sentence embeddings, where the weights are determined by an attention mechanism that learns to identify salient sentences:

(2) $$\alpha_{i}=\frac{exp(\eta (h_{i}))}{\sum_{j=1}^{N_{d}}exp(\eta (h_{j}))},\;\mathrm{where}\;\eta \left(h_{i}\right)=v_{a}^{⊤}tanh\left(W_{a}h_{i}+b_{a}\right).$$

(3) $$g=\sum_{i=1}^{N_{d}}\alpha_{i}h_{i}$$

Here, $W_{a}\in R^{d_{a}\times d_{h}}$, $b_{a}\in R^{d_{a}}$, and $v_{a}\in R^{d_{a}}$ are learnable parameters.

#### 3.4.2. SSC Loss Formulation

For a training example, let $Y^{+}=\{h_{k}^{+}{\}}_{k=1}^{M}$ be the set of embeddings of the M sentences selected into the summary. The SSC Loss $L_{\mathrm{SSC}}$ is composed of two terms:

1. Consistency Term ($L_{\mathrm{con}}$): This term minimizes the average distance between the summary sentence embeddings and the global document vector g ensuring the summary remains on-topic:
  (4) $$L_{\mathrm{con}}=\frac{1}{\left|Y^{+}\right|}\sum_{h_{k}^{+}\in Y^{+}}{∥h_{k}^{+}-g∥}_{2}$$
2. Separation Term ($L_{\mathrm{sep}}$): This term maximizes the average pairwise distance between all selected sentences, encouraging semantic diversity within the summary.
  (5) $$L_{\mathrm{sep}}=-\frac{1}{\left|Y^{+}|(|Y^{+}|-1\right)}\sum_{h_{k}^{+}\in Y^{+}}\sum_{h_{l\neq k}^{+}\in Y^{+}}{∥h_{k}^{+}-h_{l}^{+}∥}_{2}$$

Here, $h_{k}^{+}$ and $h_{l}^{+}$ denote the embeddings of two different selected sentences in $Y^{+}$, where k ≠l. Equation (5) averages the pairwise distances ${\left\|h_{k}^{+}-h_{l}^{+}\right\|}_{2}$ to encourage diversity among the selected sentences. The overall SSC loss is defined as a weighted sum of the two terms:

(6) $$L_{\mathrm{SSC}}=L_{\mathrm{con}}+L_{\mathrm{sep}}$$

The SSC objective regularizes the geometry of the selected sentence embeddings within each document. It includes two components. The consistency term pulls selected sentences toward the document-level semantic centroid g, which improves topical faithfulness. In contrast, the separation term increases pairwise distances among the selected sentences, thereby reducing redundancy. Together, these terms encourage summaries that stay on-topic while covering complementary content. In our implementation, SSC is fully differentiable and is optimized jointly with the extractive selection loss.

In this formulation, the separation term $L_{\mathrm{sep}}$ operates at the intra-document level. It regulates the local geometry of the selected sentences within the same document. Specifically, it increases pairwise distances among the selected sentence embeddings so that each sentence occupies a distinct region of the document’s semantic space. This dispersion reduces semantic overlap in the summary, while the consistency term $L_{\mathrm{con}}$ keeps the selected sentences aligned with the document’s overall theme. Accordingly, $L_{\mathrm{sep}}$ complements $L_{\mathrm{con}}$ by jointly supporting both faithfulness and local diversity. In addition, SSC constrains semantic geometry within each document, whereas the Diversity Penalty in Section 3.5 is applied at the batch level to promote global dispersion of the selected embeddings.

### 3.5. Diversity Penalty

To provide an even more direct signal against redundancy, we introduce an explicit Diversity Penalty $L_{\mathrm{div}}$. This penalty minimizes the average cosine similarity between the embeddings of all unique pairs of sentences selected in the summary for a given training batch B. Let $Y_{b}^{+}$ be the set of selected sentence embeddings for document b in the batch. The penalty for a single document is:

(7) $$L_{\mathrm{div}}^{\left(b\right)}=\frac{1}{Z}\sum_{h_{i}^{+}\in Y_{b}^{+}}\sum_{h_{j}^{+}\in Y_{b}^{+},j>i}\frac{h_{i}^{+}\cdot h_{j}^{+}}{∥h_{i}^{+}∥∥h_{j}^{+}∥}$$

Equation (7) computes pairwise cosine similarity among selected sentences within each document, normalized by the number of unique pairs (Z=|Yb+|2). The final penalty $L_{\mathrm{div}}$ is averaged across the mini batch, acting as a global regularizer that stabilizes training and mitigates redundancy patterns across documents. This design introduces only $O\left(M^{2}\right)$ cost per document, which remains negligible compared to transformer encoding:

(8) $$L_{\mathrm{div}}=\frac{1}{\left|B\right|}\sum_{b=1}^{\left|B\right|}L_{\mathrm{div}}^{\left(b\right)}$$

This training-aligned constraint differs from post hoc filtering (e.g., MMR [11]) by shaping selection behavior during learning and avoids graph construction required by [12,13].

### 3.6. Optimization

The DiCo-EXT framework is trained with a composite objective. It combines the standard binary cross-entropy loss for sentence selection with the proposed SSC loss and the diversity penalty. In addition to the SSC loss, the diversity penalty $L_{\mathrm{div}}$ reduces redundancy within each document, and it also serves as a batch-level regularizer. Within a document, $L_{\mathrm{sep}}$ shapes the geometry of the selected sentence embeddings. In each training batch, $L_{\mathrm{div}}$ helps stabilize the embedding space. It reduces cosine similarity between selected sentences from different documents. This two-level design mitigates representation collapse and promotes stable semantic dispersion. It also keeps sentence representations well separated, even in densely populated semantic regions. In this way, the diversity penalty complements the local separation loss at a broader statistical scale. The binary cross-entropy loss $L_{\mathrm{BCE}}$ is defined as:

(9) $$L_{\mathrm{BCE}}=-\frac{1}{N_{d}}\sum_{i=1}^{N_{d}}\left[y_{i}\cdot log({\hat{y}}_{i})+(1-y_{i})\cdot log(1-{\hat{y}}_{i})\right]$$

where ${\hat{y}}_{i}$ is the model’s predicted probability for sentence $s_{i}$. The final joint loss function is a weighted combination of all components:

(10) $$L_{\mathrm{total}}=L_{\mathrm{BCE}}+\beta_{\mathrm{SSC}}\cdot L_{\mathrm{SSC}}+\beta_{\mathrm{div}}\cdot L_{\mathrm{div}}$$

where $\beta_{\mathrm{SSC}}$ and $\beta_{\mathrm{div}}$ are hyperparameters that control the contribution of the SSC loss and the diversity penalty. The model is trained to minimize $L_{\mathrm{total}}$. Here, $\beta_{SSC}$ and $\beta_{div},$ respectively, control the relative contribution of intra-document and batch-level redundancy regularization.

Conceptually, the SSC loss and the Diversity Penalty operate at different geometric levels of the embedding space, and their roles are complementary. The SSC loss reshapes intra-document geometry. It pulls selected sentences toward the global semantic centroid and maintains pairwise separation, which preserves topical coherence. The Diversity Penalty focuses on inter-document dispersion across the batch. It prevents embedding collapse and encourages a stable spread of representations.

## 4. Experiments and Analysis

### 4.1. Experimental Setup

#### 4.1.1. Datasets and Evaluation Metrics

Datasets and splits. We evaluate the proposed DiCo-EXT framework on three widely used single-document summarization benchmarks: CNN/DailyMail (CNNDM), XSum, and WikiHow [31,32,33]. The CNN/DailyMail dataset consists of long-form news articles paired with multi-sentence human-written summaries. XSum contains highly abstractive and concise single-sentence summaries of news articles, representing an “extreme summarization” scenario. WikiHow includes instructional and procedural documents written in step-by-step form, which differ significantly in discourse structure and length. We adopt each dataset’s official train/validation/test partitions and refrain from any cross-domain tuning to ensure reproducibility. Together, these datasets cover diverse textual domains and summary styles, allowing a comprehensive evaluation of generalization ability.

Pre-processing. All documents are first sentence-segmented and normalized, including the removal of boilerplate or markup, punctuation unification, and preservation of the original sentence order. For efficiency, we apply uniform truncation and padding within a fixed document length cap, which affects only a small portion of samples and is held constant across all models. For extractive supervision, we generate oracle sentence labels using a greedy ROUGE-L F1 maximization against reference summaries under the same evaluation budget. At each iteration, the sentence with the largest marginal gain in ROUGE-L is added to the oracle set until the target summary length is reached. This process is identical across datasets to maintain consistency.

Evaluation metrics and rationale. To capture multiple dimensions of summary quality, we employ three complementary metrics designed to assess informativeness, redundancy, and lexical diversity:

- ROUGE-1/2/L [5]: Measures content coverage through n-gram overlap with reference summaries and remains the community standard for evaluating extractive summarization. Although ROUGE focuses on surface-level similarity, it provides a reliable proxy for overall informativeness and comparability with prior work.
- Self-BLEU [34]: Quantifies intra-summary redundancy by computing the average BLEU score of each selected sentence against all others. Lower values indicate less semantic repetition and higher diversity of information within the generated summary.
- Distinct-1/2 [35]: Calculates the proportion of unique unigrams and bigrams to assess lexical diversity. Higher Distinct scores imply broader vocabulary usage and reduced lexical overlap.

Together, these metrics form a balanced evaluation suite. ROUGE reflects the ability to retain critical information, while Self-BLEU directly measures redundancy within the summary and Distinct-n quantifies lexical variety. In our experiments, we treat CNN/DailyMail as the primary benchmark: on this dataset we report both ROUGE and diversity metrics and perform detailed analyses. XSum and WikiHow serve as generalization benchmarks with different summary styles (headline-style one-sentence abstracts and procedural instructions). On these datasets we focus on ROUGE-based comparisons to examine whether the proposed training objective transfers to other domains without re-designing the model or the evaluation protocol.

In the following experiments, we use these metrics in a targeted way. ROUGE is reported on all three datasets to evaluate overall content coverage and to compare with prior work. For diversity, we present detailed Self-BLEU and Distinct-n results and visualizations on the CNN/DailyMail corpus. This dataset contains multi-sentence news summaries, where redundancy between selected sentences is a central issue and diversity scores are easy to interpret. XSum and WikiHow have very different summary styles (headline-like one-sentence abstracts and step-by-step instructions), in which sentence-level diversity metrics are less informative. We therefore treat CNN/DailyMail as the main testbed for diversity analysis, and use the other datasets primarily to assess the generalization of ROUGE-based performance.

#### 4.1.2. Implementation Details

Our implementation is based on the BERT-based extractive summarization architecture [17] as our backbone model. The model is trained with an initial learning rate of 2 × 10^−5^ and a linear decay schedule. All models are trained on a single NVIDIA RTX 3090 GPU. The loss weighting hyperparameters are set to $\beta_{SSC}=1.0$ and $\beta_{div}=0.3$ based on validation performance. This configuration was selected to achieve an optimal balance between content coverage (ROUGE) and diversity, as detailed in our sensitivity analysis (Section 4.4).

### 4.2. Overall Performance Comparison

#### 4.2.1. Baseline Analysis

Recent advances in large language models (LLMs), such as GPT-4-turbo and Claude-3, have shown strong performance in zero-shot or instruction-based extractive summarization. However, these models operate under black-box conditions with substantial computational and financial costs, making controlled benchmarking difficult. Since DiCo-EXT aims to provide an efficient, transparent, and reproducible alternative, we restrict comparisons to publicly available extractive frameworks with accessible implementations. Nevertheless, the formulation of our objectives—jointly optimizing diversity and faithfulness—is orthogonal to model scale and can, in principle, complement LLM-based summarizers in future hybrid settings.

Table 2 presents a comprehensive comparison of overall performance across three benchmark datasets. We implement the backbone as a BERT-based extractive summarization model initialized with bert-base-uncased (≈110 M parameters). To enhance topic-sensitive sentence representations, we prepend a topic word to each sentence as an additional input token before encoding. DiCo-EXT uses the same inference architecture as the backbone and introduces only additional training objectives (SSC and Diversity Penalty), thereby incurring no architectural changes at inference time. For fair comparison, we directly report the scores from the original papers rather than reproducing the results, as the compared methods rely on heterogeneous implementations and pre-trained checkpoints that are not always publicly available. All reported numbers are taken from the official publications or their accompanying repositories.

Our proposed DiCo-EXT model shows ROUGE performance comparable to the backbone, as shown in Table 3, with only marginal differences across datasets and metrics. Specifically, on the CNNDM dataset, DiCo-EXT attains ROUGE-1, ROUGE-2, and ROUGE-L scores of 43.32, 20.45, and 39.75, reflecting slight but stable gains over the backbone model. On WikiHow, our method achieves ROUGE-1/2/L of 30.18, 8.48, and 27.95, showing comparable ROUGE performance in a different domain. For XSum, DiCo-EXT achieves ROUGE-1/2/L of 25.38/5.25/21.29, with marginal improvements over the backbone while preserving the concise style required by this dataset.

This consistent performance across diverse datasets (from news to instructional texts) underscores the generalizability of our approach. The key insight from these results is that our approach effectively addresses the redundancy problem—a major limitation of conventional extractive summarizers—without degrading the fundamental content coverage measured by ROUGE. DiCo-EXT improves diversity and reduces redundancy while keeping ROUGE performance comparable. The subsequent diversity analysis will quantify redundancy and diversity directly, showing that the main gains of DiCo-EXT lie in diversity improvement rather than ROUGE increases. This indicates the potential of explicitly optimizing diversity alongside content selection in extractive summarization.

#### 4.2.2. Diversity Analysis

We focus our diversity analysis on the CNN/DailyMail dataset, where summaries consist of multiple sentences and redundancy between selected sentences is most evident. The diversity analysis in Table 3 reveals substantial improvements on this corpus. DiCo-EXT reduces Self-BLEU from 0.86 to 0.72 and increases Distinct-2 from 0.61 to 0.78. These results indicate that our method effectively addresses the redundancy problem in extractive summarization, producing more diverse and lexically rich summaries.

**Figure 2:**
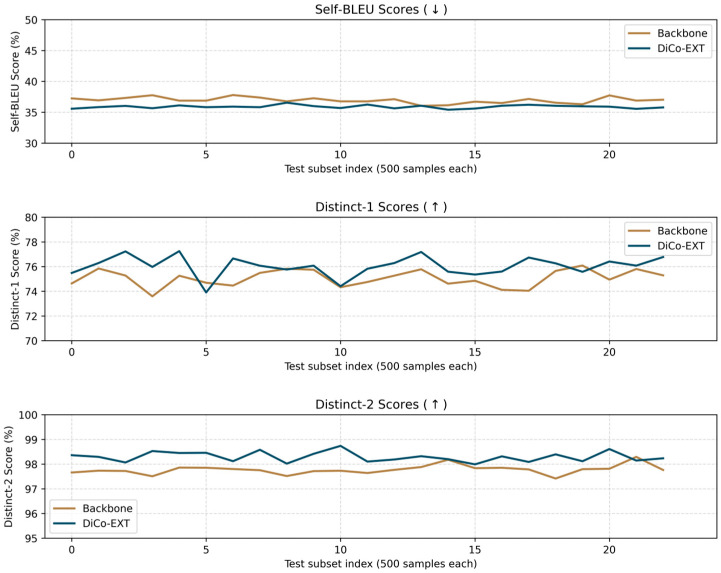
Diversity metrics on CNNDM: backbone vs. DiCoEXT.

These changes indicate that the summaries contain less repeated content and more varied expressions. The main benefit of our method is thus not a large ROUGE increase, but a better use of each summary sentence: more unique information is conveyed with fewer overlaps. Readers can obtain similar key facts with less redundant text. This effect is captured by the diversity metrics and suggests that small differences in ROUGE may hide noticeable improvements in readability and information efficiency. Figure 2 visually confirms the diversity improvements achieved by DiCo-EXT on the CNNDM dataset. Each lines reports the average over 500 test samples. The consistent pattern of lower Self-BLEU and higher Distinct scores across multiple test batches demonstrates the robustness of our approach. The improvements are particularly notable in the Distinct-2 metric, which measures bigram diversity and is a stronger indicator of lexical richness than unigram-based metrics.

### 4.3. Component Ablation Study

To better understand the contribution of each component in our proposed framework, we conduct a comprehensive ablation study on the CNNDM dataset. The results are presented in Table 4.

The ablation study in Table 4 helps clarify the role of each component. Adding only the SSC loss improves both ROUGE and diversity: Self-BLEU drops from 0.86 to 0.79, and Distinct-2 rises from 0.61 to 0.69. This suggests that the semantic consistency constraint is important for reducing redundancy while keeping relevant content. Adding only the Diversity Penalty also improves diversity, though the effect on ROUGE is slightly smaller. When both components are enabled, the full DiCo-EXT model achieves the best overall performance. ROUGE scores are the highest, and the summary is the least redundant, with Self-BLEU reduced to 0.72 and Distinct-2 increased to 0.78. In this setting, SSC keeps sentences aligned with the main themes of the document, and the Diversity Penalty discourages the selection of sentences that carry very similar information. The model is therefore encouraged to choose sentences that complement each other and cover different aspects of the document.

Although DiCo-EXT introduces two additional objectives, the overall computational complexity remains comparable to standard BERT-based extractive models. The SSC module operates on sentence embeddings within each document and involves only pairwise distance computations among the selected sentences, resulting in an $O\left(M^{2}\right)$ cost where M is the number of selected sentences. Similarly, the Diversity Penalty computes cosine similarities within each document and performs a lightweight batch-level averaging, without requiring any external structures or graph construction. Since both operations are implemented on low-dimensional sentence embeddings and are independent of the encoder sequence length, their overhead is negligible relative to the Transformer encoding process. Moreover, DiCo-EXT does not introduce additional trainable parameters beyond the linear projection layer, preserving the compactness and reproducibility of the baseline architecture.

Beyond computational efficiency, the observed improvements in diversity metrics are not merely statistical artifacts but reflect a structural shift in how the model organizes semantic information during selection. By introducing the SSC constraint, the model learns to anchor sentence representations around distinct thematic regions of the document, effectively reducing representational overlap in the embedding space. Meanwhile, the Diversity Penalty encourages dispersion across these regions, preventing the model from converging toward redundant local optima. Consequently, DiCo-EXT produces summaries that are not only less repetitive but also more semantically comprehensive, as each selected sentence contributes unique contextual information to the overall narrative.

### 4.4. Hyperparameter Sensitivity Analysis

We further investigate the sensitivity of our model to key hyperparameters, specifically the loss weighting factors $\beta_{\mathrm{SSC}}$ and $\beta_{\mathrm{div}}$. The results are shown in Table 5.

The hyperparameter sensitivity analysis in Table 5 reveals several important patterns: First, the model performance shows reasonable stability across different hyperparameter settings, with ROUGE scores varying within a narrow range (43.15–43.32 for ROUGE-1) across all configurations. This suggests that our approach is relatively robust to the exact weighting of the loss components.

Second, the optimal balance between content selection quality (as measured by ROUGE) and diversity is achieved with $\beta_{\mathrm{SSC}}=1.0$ and $\beta_{\mathrm{div}}=0.3$. This configuration produces the best ROUGE scores while maintaining strong diversity metrics.

Third, we observe a clear trade-off between content coverage and diversity when adjusting the hyperparameters. Increasing $\beta_{\mathrm{div}}$ beyond 0.3 leads to further improvements in diversity metrics (Self-BLEU decreases to 0.69 and 0.67, while Distinct-2 increases to 0.81 and 0.82) but at the cost of slightly reduced ROUGE scores. Similarly, increasing $\beta_{\mathrm{SSC}}$ to 1.5 improves diversity but slightly reduces ROUGE scores.

**Figure 3:**
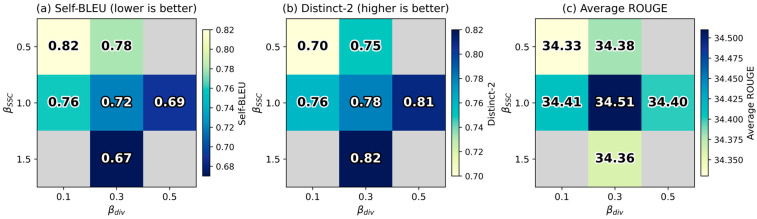
Visualization of hyperparameter sensitivity with respect to $\beta_{SSC}\;$ and $\beta_{div}$.

Figure 3 provides a visual summary of how different settings of $\beta_{SSC}$ and $\beta_{div}$ influence the three evaluation metrics. Gray cells indicate configurations that were not evaluated. The heatmaps reveal several clear patterns. The row with $\beta_{SSC}=1.0$ shows relatively consistent colors across all three panels, indicating stable behavior under this setting. In contrast, increases in $\beta_{div}$ shift the color toward the extremes in the Self-BLEU and Distinct-2 plots, showing stronger effects on redundancy and diversity. The combination $\beta_{SSC}=1.0$ and $\beta_{div}=0.3$ forms the most balanced region in the figure, with competitive ROUGE and reasonable diversity. The gray cells mark configurations that were not included in the study and help to clarify the coverage of the search space. This trade-off suggests that while both components contribute to reducing redundancy, excessive emphasis on either component may slightly compromise content coverage. The optimal configuration balances these competing objectives to produce summaries that are both comprehensive and diverse.

### 4.5. Case Study

Table 6 presents a qualitative comparison that illustrates the different sentence selection strategies of the backbone model and our DiCo-EXT approach. The backbone model selects sentences (1, 2, and 5), which are all highly scored when viewed in isolation. However, these sentences focus on the same part of the story: the announcement itself and the company’s claim that the initiative is ambitious. This selection pattern leads to a summary with repeated emphasis on the announcement, but with limited coverage of concrete plans or external reactions. The ROUGE-1 score is relatively high (43.20) because these sentences share many surface n-grams with the reference summary, yet much of this overlap comes from similar phrases describing the same fact.

In contrast, DiCo-EXT selects sentences (2, 4, and 6). Sentence (2) describes the emission reduction goal, sentence (4) provides specific implementation details, and sentence (6) reports analysts’ reactions and concerns. Each sentence brings in a different aspect of the event. The resulting summary offers a clearer picture of what the initiative aims to achieve, how it will be implemented, and how external experts respond. Although the ROUGE-1 score is slightly lower (41.8), the Self-BLEU score drops from 0.84 to 0.71, which means that the selected sentences are less similar to one another. The user sees less repetition and gains more unique information per sentence.

This example also connects the case study with the quantitative diversity results. The reduction in Self-BLEU and the more balanced coverage of goals, plans, and reactions match the trends observed in Table 3 and the ablation study. The SSC loss encourages the model to keep each selected sentence aligned with the main topic of the document, so DiCo-EXT still chooses sentences that are clearly relevant. The diversity penalty then discourages the model from picking multiple sentences that say almost the same thing. Together, these objectives guide the model toward summaries where each sentence plays a distinct role. In practice, this pattern appears in many CNN/DailyMail examples. The backbone model often prefers several strong but similar sentences, whereas DiCo-EXT tends to mix a core fact with complementary details and viewpoints. This case study therefore illustrates how a small difference in ROUGE can correspond to a noticeable improvement in redundancy and information coverage, and why diversity-oriented training is useful even when standard overlap-based scores remain close.

## 5. Conclusions

This paper presented DiCo-EXT, an extractive summarization framework that optimizes semantic faithfulness and diversity within a single learning objective. Instead of evaluating sentences in isolation under a ROUGE-driven objective, DiCo-EXT shapes the selected set through two components. The SSC module aligns selected sentences with a document-level representation and encourages separation among them, which helps maintain topical coherence and reduce redundancy. The Diversity Penalty extends this idea to the batch level by discouraging highly similar embeddings across documents and stabilizing the representation space.

Experiments on three benchmarks show that DiCo-EXT improves diversity metrics such as Self-BLEU and Distinct-n while keeping ROUGE competitive. This supports the view that faithfulness and diversity can be improved together through simple regularization terms, without changing the underlying model architecture. While large generative models such as BART-based systems or ChatGPT (gpt-3.5-turbo) variants have advanced abstractive summarization, extractive models remain attractive in domains that require verifiable summaries. Our results suggest that redundancy-aware training objectives are a practical way to strengthen such models. In future work, we will examine how DiCo-EXT can complement LLM-based and hybrid summarization systems. In extract-then-abstract pipelines, DiCo-EXT can act as a redundancy-aware extractor that provides a compact and diverse evidence set for downstream LLM rewriting. We will also study retrieval-augmented summarization, where redundancy-aware selection may improve context utilization under a fixed token budget. Beyond integration, LLMs may provide pseudo-labels or preference signals to train efficient extractive models, while our SSC and diversity terms offer lightweight, fully differentiable objectives to stabilize optimization and control redundancy. Finally, we will explore adaptive, document-dependent weighting of these terms: instead of fixed coefficients, $\beta_{SSC}$ and $\beta_{div}$ could be adjusted based on document length or an estimated redundancy score (e.g., average semantic similarity among candidate sentences), strengthening redundancy control only when needed.

## Figures and Tables

**Table 1:** Description of the principal mathematical symbols used in Section 3.

Symbol	Description
D	The input document.
$s_{i}$	The i-th sentence in the document.
$h_{i}$	Semantic embedding vector of sentence $s_{i}$.
Y	The final extracted summary, a set of sentences.
$Y^{+}$	Set of embeddings of sentences selected for the summary.
g	Global semantic representation vector of the entire document.
$L_{\mathrm{SSC}}$	Semantic Similarity Consistency loss.
$L_{\mathrm{con}}$	Consistency term of $L_{\mathrm{SSC}}$, pulls summary towards g.
$L_{\mathrm{sep}}$	Separation term of $L_{\mathrm{SSC}}$, pushes selected sentences apart.
$L_{\mathrm{div}}$	Diversity Penalty loss, minimizes cosine similarity within $Y^{+}$.
$L_{\mathrm{BCE}}$	Binary Cross-Entropy loss for sentence selection.

**Table 2:** ROUGE results on CNN/DailyMail, WikiHow, and XSum for the BERT-based backbone (bert-base-uncased with a prepended topic word per sentence) and DiCo-EXT. (-) indicates that the corresponding paper did not report results for that dataset.

	CNNDM			WikiHow			XSum		
Model	R-1	R-2	R-L	R-1	R-2	R-L	R-1	R-2	R-L
Oracle	52.59	31.24	48.87	39.80	14.85	36.90	29.79	8.81	22.66
RFAR [36]	40.64	17.49	36.01	27.38	6.02	25.37	-	-	-
FAR [36]	40.83	17.85	36.91	27.54	6.17	25.46	-	-	-
PACSUM [37]	40.7	17.8	36.9	-	-	-	-	-	-
LLCS [38]	40.92	17.88	37.27	-	-	-	-	-	-
ChatGPT-EXT (gpt-3.5-turbo) [39]	39.25	17.09	25.64	-	-	-	19.85	2.96	13.29
AES-REP [40]	43.21	19.90	39.38	29.46	7.75	27.23	-	-	-
BackBone [17]	43.11	20.23	39.54	29.91	8.32	27.76	25.05	5.17	21.03
DiCo-EXT	**43.32**	**20.45**	**39.75**	**30.18**	**8.48**	**27.95**	**25.38**	**5.25**	**21.29**

**Table 3:** Diversity metrics comparison on CNNDM dataset. Lower Self-BLEU and higher Distinct scores indicate better diversity.

Model	Self-BLEU	Distinct-1	Distinct-2	Diversity Gain
Backbone	0.86	0.38	0.61	-
DiCo-EXT (Ours)	0.72	0.52	0.78	+27.9%

**Table 4:** Ablation study of DiCo-EXT components on CNNDM dataset.

Model Variant	R-1	R-2	R-L	Self-BLEU	Distinct-1	Distinct-2
Backbone (BCE only)	43.11	20.23	39.54	0.86	0.38	0.61
+$L_{\mathrm{SSC}}$ only	43.19	20.31	39.62	0.79	0.45	0.69
+$L_{\mathrm{div}}$ only	43.15	20.27	39.58	0.81	0.43	0.66
DiCo-EXT (Full)	**43.32**	**20.45**	**39.75**	**0.72**	**0.52**	**0.78**

**Table 5:** Hyperparameter sensitivity analysis on CNNDM dataset. $\beta_{SSC}$ controls local geometric separation; $\beta_{div}$ controls global diversity regularization.

$\beta_{\mathrm{SSC}}$	$\beta_{\mathrm{div}}$	R-1	R-2	R-L	Self-BLEU	Distinct-2
0.5	0.1	43.15	20.26	39.57	0.82	0.70
0.5	0.3	43.20	20.32	39.63	0.78	0.75
1.0	0.1	43.22	20.35	39.65	0.76	0.76
1.0	0.3	43.32	20.45	39.75	0.72	0.78
1.0	0.5	43.23	20.34	39.64	0.69	0.81
1.5	0.3	43.18	20.30	39.60	0.67	0.82

**Table 6:** Qualitative comparison between the backbone model and DiCo-EXT on a sample from the CNNDM test set.

Source Document (Excerpt):
“(1) The company announced a new environmental initiative on Monday. (2) The initiative aims to reduce carbon emissions by 50% by 2030. (3) CEO John Smith emphasized the company’s commitment to sustainability. (4) The plan includes investments in renewable energy and electric vehicle infrastructure. (5) Smith stated that this initiative represents their most ambitious climate goal to date. (6) Analysts have praised the move but question the feasibility of the timeline.”
Backbone Model Summary:
(1) The company announced a new environmental initiative on Monday.
(2) The initiative aims to reduce carbon emissions by 50% by 2030.
(5) Smith stated that this initiative represents their most ambitious climate goal to date.
ROUGE-1: 43.20, Self-BLEU: 0.84
Analysis: Selected sentences focus on announcement details but lack breadth, resulting in redundancy.
DiCo-EXT Summary:
(2) The initiative aims to reduce carbon emissions by 50% by 2030.
(4) The plan includes investments in renewable energy and electric vehicle infrastructure.
(6) Analysts have praised the move but question the feasibility of the timeline.
ROUGE-1: 41.8, Self-BLEU: 0.71
Analysis: Covers diverse aspects including the goal, specific implementation plans, and expert analysis, providing a more comprehensive summary.

## Data Availability

We utilize three benchmark datasets for our experiments: the CNN/DailyMail reading comprehension dataset [31], and the XSum [32] and WikiHow [33] summarization datasets. CNN/DailyMail: https://huggingface.co/datasets/ccdv/cnn_dailymail; XSum: https://github.com/EdinburghNLP/XSum; WikiHow: https://github.com/HiDhineshRaja/WikiHow-Dataset (all accessed on 1 December 2025).
